# Medicaid Eligibility and Time to Re-incarceration Among Previously Incarcerated Subjects With Schizophrenia

**DOI:** 10.36469/9845

**Published:** 2016-10-31

**Authors:** Chris Kozma, Michael Dickson, Jacqueline Pesa, Carmela J. Benson

**Affiliations:** 1 CK Consulting Associates, LLC, Saint Helena Island, SC; 2 University of South Carolina College of Pharmacy, Columbia, SC; 3 Janssen Scientific Affairs, LLC, Titusville, NJ

**Keywords:** eligibility, medicaid, re-incarceration, incarceration, schizophrenia

## Abstract

**Background:** Many persons with severe mental illness qualify for Medicaid coverage. However, under federal law, states must either suspend or terminate eligibility once they are incarcerated. We hypothesize that prompt re-acquisition of Medicaid eligibility following release from incarceration lowers the risk of re-incarceration.

**Objective:** To assess the relationship between Medicaid eligibility and risk of re-incarceration among previously incarcerated schizophrenia diagnosed subjects.

**Methods:** Study subjects were selected between January 1, 2006 and September 30, 2011 from a single state Medicaid database that was combined with department of corrections data. Subjects were included if they had a schizophrenia diagnosis (International Classification of Diseases, 9th Revision, Clinical Modification [ICD- 9-CM] code 295.xx), were between the ages of 18 and 62, and had been released from incarceration. Covariates included age, race, gender, marital status, and reason for incarceration. Time to Medicaid eligibility after release from incarceration, cumulative days of eligibility, and whether they were eligible on the re-incarceration date were evaluated in independent models. One and three-year Cox Regression models analyses (p<0.05) were used to evaluate the hazard for re-incarceration.

**Results:** The 932 subjects were 26.5% white, 73.7% male and were, on average, 37.6 years old on their index date (i.e., incarceration release date). They were 73.5% single or divorced and 12.7% were incarcerated for a substance abuse violation. In the 1-year follow-up period, 110 subjects (11.8%) were re-incarcerated. In the 3-year follow-up period 209 (22.4%) were re-incarcerated. Age (in years) was the only significant predictor of re-incarceration for the 1-year models (hazard ratio [HR]=0.976; confidence interval [CI]=0.957, 0.994). Eligibility was a significant predictor in the 3-year follow-up models. A longer ‘time to first eligibility’ (HR=1.046; CI=1.017, 1.075 was associated with a greater hazard for re-incarceration. Being eligible at the time of re-incarceration (HR=0.659; CI=0.498, 0.870) was associated with a lower hazard, and the cumulative number of months of eligibility (HR=0.978; CI=0.958, 0.997) and age were associated with a lower hazard for re-incarceration (HR=0.986; CI=0.973, 0.999).

**Conclusions:** Access to Medicaid health services post-release may reduce the risk of re-incarceration.

## BACKGROUND

In the United States, the majority of states have a jail or prison system that houses more individuals with serious mental illness (SMI) than the state's largest mental hospital.[1] Mental illness is astoundingly prevalent among the incarcerated population, with one study estimating that 64% of the jailed population in the United States has some form of mental illness[2], while another reported SMI in nearly 15% of incarcerated males and 31% of females[3]; a rate 3-6 times that of the general population.[4] As Lamb and Weinberger stated in a 1998 publication, the prevalence of severely mentally ill persons in jails and prisons is an "urgent problem".[5]

Recidivism is high in the SMI population and is a major cost burden to the criminal justice system at the local and national levels. Lovell and colleagues found that 64% of mentally ill offenders were re-arrested within 18 months of release.[6] Baillargeon et al report similar findings, that inmates with major psychiatric disorders (major depressive disorder, bipolar disorder, schizophrenia, and non-schizophrenic psychotic disorders) have substantially increased risk of multiple incarcerations over a 6-year period.[7] Efforts to uncover risk factors for incarceration among the SMI population include a variety of socioeconomic factors, including de-institutionalization, lack of adequate community supports, difficulty gaining access to community treatment and attitudes of law enforcement and society. The National Comorbidity Survey Replication suggested additional risk factors including male gender, past receipt of welfare payments, one or more weeks of homelessness, and history of substance abuse or a dependence diagnosis.[8] Similar risk factors have been identified for re-incarceration in this population, with homelessness, substance abuse, lack of health insurance, and lack of timely receipt of outpatient or case management services post-release described as modifiable.[9] Additional evidence comes from a study of New York Medicaid beneficiaries which found that schizophrenia patients in hospitals, homeless shelters, and prisons have an estimated 60% re-admission rate, a higher risk of arrests and incarcerations, and are less likely to have health insurance coverage compared with the general population.[10]

The role of Medicaid access in mitigating the risk of re-incarceration for the SMI population has been investigated by Morrissey and colleagues in a study of this population in four counties in Washington State and Florida.[11] The unit of analysis for this study was detentions rather than individual subjects. The analysis included 45.7% schizophrenia detentions with over 51% for subjects with affective disorders. The authors examined the difference between subjects with Medicaid eligibility at the time of release from incarceration and subjects without Medicaid eligibility upon release from incarceration and followed all subjects for 90 days. In general, they found that detentions with Medicaid eligibility upon release from incarceration had a shorter time to first use of mental health services, and more days of service than subjects who did not have Medicaid eligibility upon release, The Morrissey et al study did not examine time to re-incarceration as related to Medicaid eligibility at the time of release, nor did it examine the outcomes related to differing times to obtain Medicaid eligibility.

The study reported here examines the effect of time to Medicaid eligibility on re-incarceration by three parameters: 1) Medicaid eligibility at the time of re-incarceration (yes/no), 2) cumulative months of Medicaid eligibility post-release, and 3) elapsed time from release to date of Medicaid eligibility. These endpoints were studied for two time periods (1 year and 3 years post-release from incarceration). The current study focuses only on individuals with schizophrenia. This study expands on the Morrissey et al work to further explicate the salutary effect of Medicaid on re-incarceration rates for the SMI population. The sources cited here as well as others provide an abundance of evidence that schizophrenia patients require social and health care support services post-release to mitigate the risk of re-incarceration.

## OBJECTIVES

The literature on incarceration among the SMI population shows a substantial relationship between a lack of access to healthcare services and re-incarceration. Given that this population relies heavily on public programs such as Medicaid, the intent of the current study is to investigate the effect of time to acquire Medicaid eligibility on the risk for re-incarceration among individuals diagnosed with schizophrenia.

## METHODS

### Data Source and Subject Selection

Data for the study were provided by the South Carolina (SC) Office of Research and Statistics (ORS). The initial data extraction by ORS included all subjects with a Medicaid claim containing a schizophrenia ICD9-CM diagnosis (295.xx) or an antipsychotic claim (i.e., pharmacy claim or HCPCS on a medical claim) between January 1, 2005 and September 30, 2012 that also had at least one incarceration record in the SC criminal justice system. For individuals meeting this criterion, complete data were retrieved for Medicaid claims (hospital, ambulatory, nursing home, and pharmacy), Medicaid eligibility records with subject demographics, SC Law Enforcement Division (SLED) records, and SC Department of Corrections (DOC) records. Medicaid subjects were selected for inclusion in the study if they had at least one medical (hospital or ambulatory) claim with a schizophrenia diagnosis (International Classification of Diseases 9th Revision Clinical Modification [ICD-9-CM] code 295.xx) between January 1, 2005 and September 30, 2011; were released from incarceration in the SC prison system between January 1, 2006 and September 30, 2011 (subject identification period) and were ≥18 and ≤62 years of age on the date of their first release from incarceration in the selection period. Each subject's index date was the most recent release from incarceration in the selection period. The study time line with three patient examples is shown in Figure 1.

**Figure 1.** Study Diagram

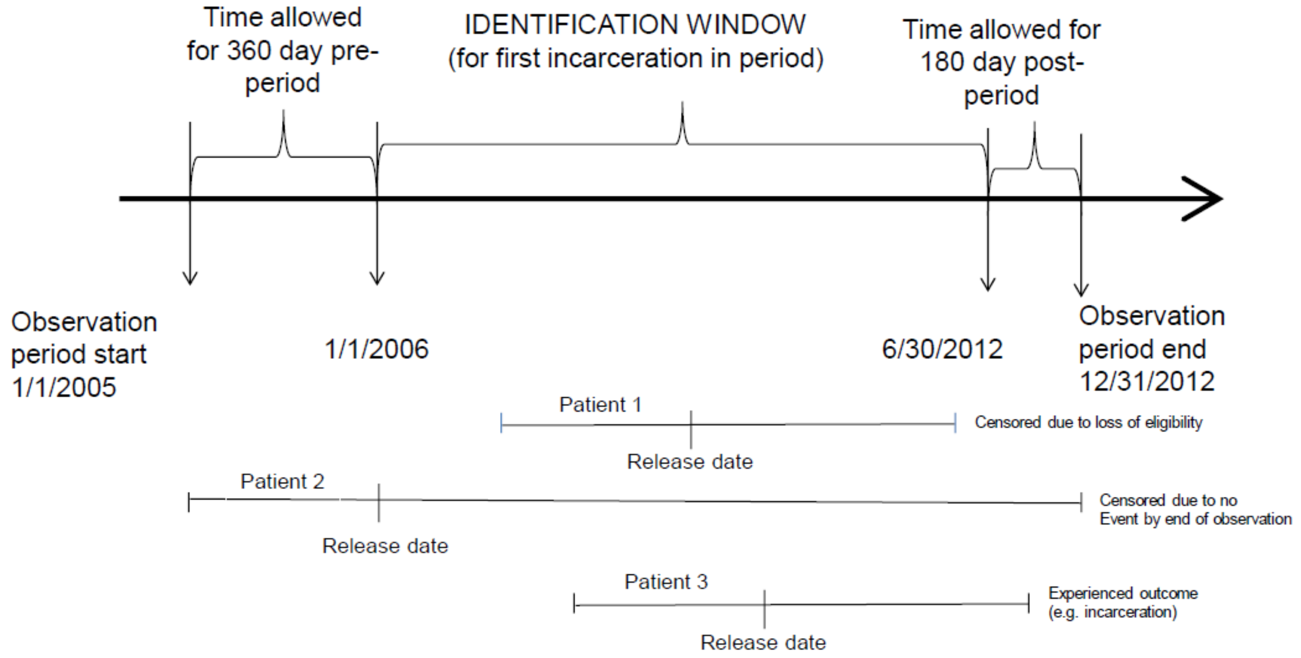

Note: Patient data were assessed from date of release until the end of the study period or until loss of eligibility.

### Statistical Analysis

Cox regression models using up to 1 and 3 years of follow-up beginning with the first release from incarceration were used to evaluate the hazard for re-incarceration (hazard ratios [HRs] with 95% confidence intervals [CIs]). Three independent models were assessed for each of three Medicaid eligibility variables plus covariates including: age at release on the index date, gender, race (white, non-white), marital status (married, single/ divorced) and reason for incarceration (e.g., substance abuse-related, other). The three eligibility measures assessed were:

Eligibility Measure 1: Eligibility at the time of re-incarceration
Eligibility Measure 2: Cumulative months of eligibility
Eligibility Measure 3: Quarters of time to the first date of eligibility

The eligibility variables were treated as time-dependent variables (i.e., allowing for changes in Medicaid eligibility over time). For example, patients could accumulate days of eligibility over time as opposed to defining the variable as static (i.e., measured at one point in time). For the analysis Indicator variables were created for presence of Medicaid eligibility (yes/no) for each post-index day and counters for the number of days (expressed as quarters) until eligibility. This time-dependent variable approach allowed assessment of eligibility at time points when re-incarceration occurred.[12]

The three different eligibility measures address the following questions:

- Does eligibility at the point of re-incarceration affect the risk for re-incarceration (eligibility measure 1)?
- Does the total number of months a subject is eligible after release affect the risk for re-incarceration (eligibility measure 2)?
- Does the duration of time (in quarters) that it takes a subject to become eligible post-release affect the risk for re- incarceration (eligibility measure 3)?

A total of five models were evaluated for two time periods (1 and 3 years) for all three eligibility measures, however, eligibility measure 3 was not evaluated in a 1-year model due to the short time frame. No adjustment was made for multiplicity. All analyses were performed using SAS for Windows 9.3 (Cary, NC).

## RESULTS

Subject Characteristics
Table 1 presents the demographic characteristics for the 932 subjects who met the selection criteria. The average age was 37.6 years and most of the subjects were male (73.7%), non-white (73.5%), either single or divorced (73.5%) and 12.7% were incarcerated for substance abuse-related reasons. In the 1 year following release, 11.8% were re-incarcerated and 22.4% were re-incarcerated by the end of 3 years.

### Regression Results

Each of the Cox regression models predicting time to re-incarceration included the same set of covariates; only the eligibility variable was different for each model. Covariates, other than eligibility, included in the models were age at index date, marital status, gender, and race (white vs. non-white). Models that included a variety of interaction terms were examined but none of the interactions were statistically significant.

**Table 1.** Baseline Subject Characteristics (n=932)

| | | |
|---|---|---|
| Age, years, mean (SD) | 37.6 | (10.3) |
| Sex, n (%) | | |
| Male | 687 | (73.7) |
| Female | 245 | (26.3) |
| Race, n (%) | | |
| White | 247 | (26.5) |
| Black or African-American | 605 | (64.9) |
| Other | 80 | (8.6) |
| Marital Status, n (%) | | |
| Single/Divorced | 685 | (73.5) |
| Married | 77 | (8.3) |
| Other | 170 | (18.2) |
| Reason for incarceration, n (%) | | |
| Substance abuse-related | 118 | (12.7) |
| Other | 814 | (87.3) |
| Re-incarceration, n (%) | | |
| At 1 year | 110 | (11.8) |
| At 3 years | 209 | (22.4) |

SD: standard deviation

### One-year Follow-up Models

Age was significant in the one-year follow-up models. For eligibility measure 1 (eligibility at time of reincarceration) each additional year of age was associated with a 2.4% lower hazard for incarceration (HR=0.976; 95% CI: 0.957, 0.994; p=0.0104). Results were almost identical for age in the cumulative months of eligibility (eligibility measure 2) model (HR 0.976; 95% CI: 0.957, 0.994; p=0.0101). Eligibility measure 3 (quarters of time to the first date of eligibility) was not evaluated in the 1-year follow-up due to the short follow-up time.

### Three-year Follow-up Models (Figures 2-4)

Age and Medicaid eligibility were significant in each of the three-year follow-up models. For age, the HRs were similar to the 1-year models, ranging from 0.986 to 0.988, all indicating reduced hazard for reincarceration with each additional year of age at the index date (1.2% to 1.4% lower hazard). Hazard ratios for Medicaid eligibility at the time of re-incarceration showed 34.1% lower odds of re-incarceration if the subject was eligible at the time of re-incarceration (eligibility 1: HR 0.659; 95% CI 0.498, 0.870; p=0.0033). The cumulative number of months of eligibility model (eligibility 2) showed a 2.2% lower hazard for reincarceration for each additional month of cumulative eligibility (HR 0.978; 95% CI 0.958, 0.997; p=0.0274). Cumulative number of quarters until the first eligibility (i.e., delay in eligibility) was associated with a greater hazard for incarceration of 4.6% (HR 1.046; 95% CI 1.017, 1.075; p=0.0016).

**Figure 2.** Three-year Survival Analysis Hazard Ratios and 95% Confidence Intervals for Eligibility at Re-incarceration (Eligibility Measure 1)

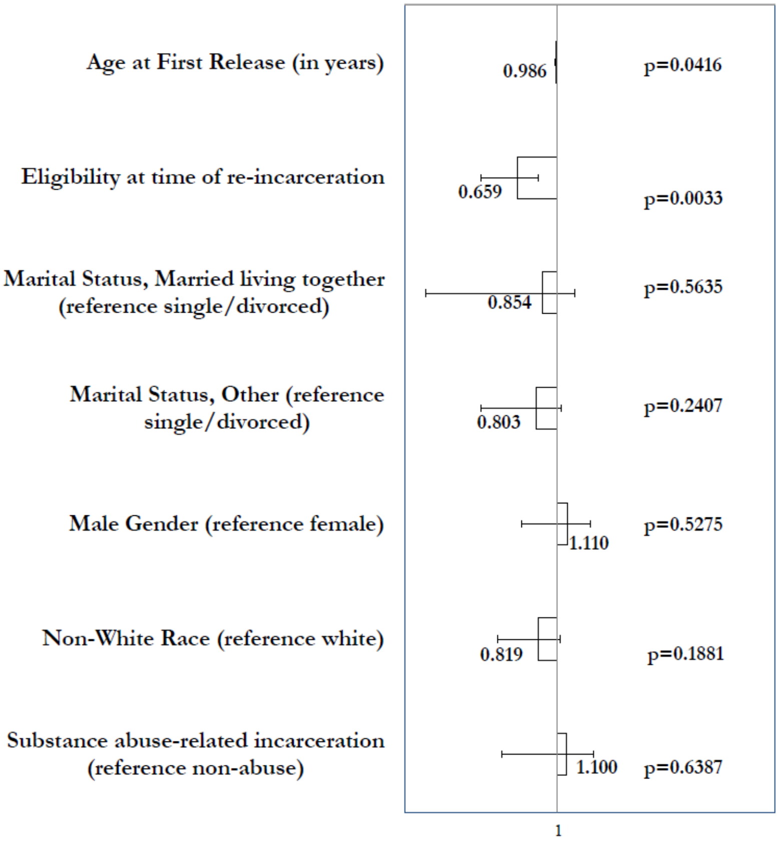

**Figure 3.** Three-year Survival Analysis Hazard Ratios and 95% Confidence Intervals for Eligibility at Re-incarceration (Eligibility Measure 2)

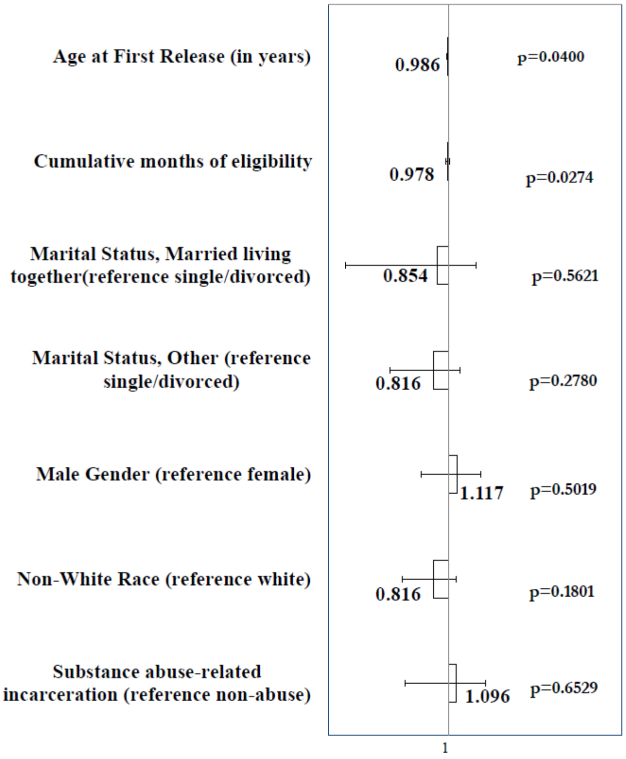

**Figure 4.** Three-year Survival Analysis Hazard Ratios and 95% Confidence Intervals for Eligibility at Re-incarceration (Eligibility Measure 3)

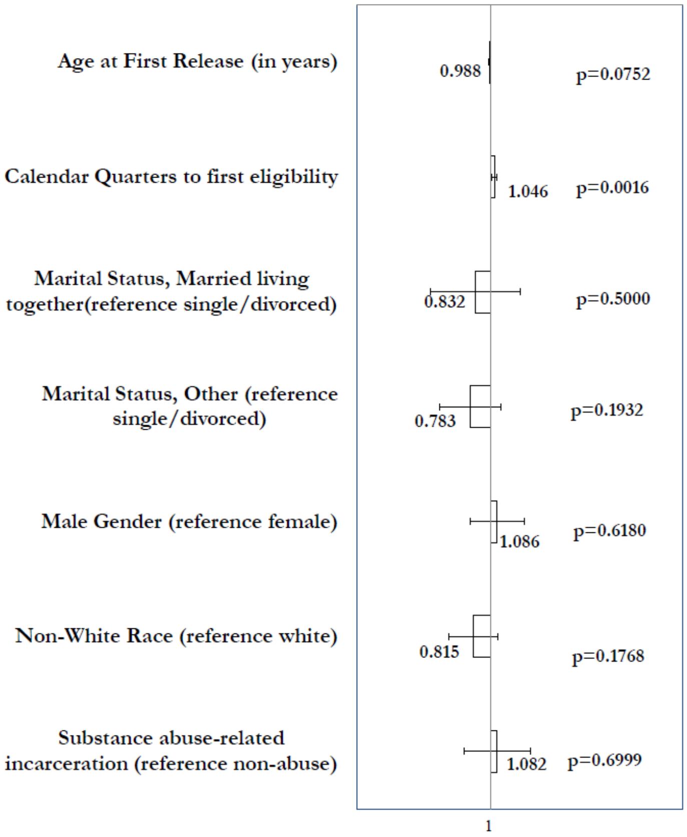

## LIMITATIONS

The limitations for this study include those of any study based on retrospective claims data, including the possibility of coding errors. Merging data from a variety of sources presents challenges. While the encrypted unique subject identifiers are considered equivalent across data sources, there is no assurance that other variables would be defined in a consistent manner across databases. We addressed this potential problem by using all demographic variables from the Medicaid eligibility file.

It was assumed that patient's incarceration status would be known for the entire 1 or 3 year observation period (i.e., censoring occurred at the end of observation). It was not possible to determine if patients were incarcerated in a state other than SC. The incarceration data came from the SC Department of Corrections and most likely reflects releases only from SC prisons.

Persons that were incarcerated in local jails and time spent in local jails may not be reflected in total incarceration time. It is possible that actual incarceration rates could be greater. It was also not possible to identify deaths; however given the relatively short time frame for the models it seems unlikely that this would be a significant factor.

The 1-year models did not show a significant relationship between eligibility and the hazard for reincarceration. It is possible that there might be unmeasured factors that are more relevant for near-term re-incarceration. It is also possible that the number of events within eligibility categories was too small for detecting effects. More research using different techniques and larger samples is warranted for evaluation of near-term effects of eligibility on the hazard for re-incarceration.

Limitations are also imposed by the absence of some variables that the literature suggests would be important for estimating the hazard for re-incarceration. For example, there was a "type of crime" variable available in the records, but it was found to be problematic because of non-standard reporting. Clinical measures and an indicator of severity also were not available. Comorbidity was measured as the number of diagnoses for mental health issues (including depression, substance abuse, alcohol dependence, personality disorders, and depression) but was not a significant variable in the final models.

## DISCUSSION

The high prevalence rate for incarcerated persons with SMI and lack of attention to the risk factors known to contribute to a high rate of re-incarceration have created a costly situation for states and local governments who pay the greatest share of the costs for incarcerated persons with SMI. Cost shifting of mental health services from care facilities to the criminal justice system is occurring due to the likelihood that persons with SMI will be incarcerated[13] and receive psychiatric inpatient treatment while incarcerated.[14] Contributing to the cost and complexity of care for incarcerated persons with SMI is the chronic nature of the illness and the need for ongoing treatment and transition planning upon release. After release from incarceration, barriers to healthcare access often lead to noncompliance with treatments due to the financial barriers for obtaining health insurance coverage. To help patients with schizophrenia successfully re-enter community life and better fulfill their potential, their complex treatment needs must be understood.

Results from the 3-year models in this study support the hypothesis that re-incarceration of individuals with schizophrenia is associated with a lack of timely acquisition of health care coverage through Medicaid eligibility. These findings are consistent with a study by Morrissey et al. conducted in the state of Washington that found that individuals with SMI referred for expedited Medicaid restoration had a 36% higher probability of receiving Medicaid coverage in the first 12 months following release from incarceration than those not referred.[15] This was associated with a higher rate of commonly needed services such as use of antipsychotic and antidepressant medications, alcohol and drug treatment, and outpatient mental health services. An earlier study by Morrissey et al examined the use of Medicaid services for SMI persons released from incarceration in King County Washington and Pinellas county Florida using a prospective cohort design.[16] Persons with Medicaid coverage were more likely to use healthcare services in the first 90 days after release than those without Medicaid coverage. Both groups with Medicaid coverage had shorter times to followup (5.3 days sooner in Washington and 6.8 days sooner in Florida) than those without Medicaid coverage.

Many people with SMI have Medicaid eligibility while in the general population, but most lose eligibility when incarcerated. At the time of this analysis only 14 states have policies that allow Medicaid coverage for some eligible inmate patient care.[17] This partial coverage occurs if the Medicaid eligible inmate is an inpatient at a community-based facility (e.g., inpatient hospital, nursing facility, juvenile psychiatric facility, intermediate care facility, etc., the "inmate exclusion provision") for at least 24 hours.[18] Rosen et al surveyed state prison system policies (42 of 50 systems responded) and found that upon incarceration Medicaid eligibility is either suspended (9 systems, 21.4%) or terminated (28 systems, 66.7%).[19] Suspension of Medicaid eligibility allows an inmate to remain on Medicaid rolls but will have only qualified inpatient services covered by Medicaid. Termination of Medicaid eligibility requires an individual to repeat the eligibility determination process which can take 45 - 90 days. Tobler, among others, has recommended that Medicaid eligibility be suspended upon incarceration, rather than terminated to facilitate coverage upon parole.[20] Support for this policy is provided by a study in two Florida counties reporting that having Medicaid coverage when released from incarceration is associated with fewer subsequent detentions.[21]

Transition planning/continuity of care planning from incarceration to the community are a critical component of successful re-entry to the community. Fontanarosa, et al, conducted a comprehensive review of serious mentally ill patients (defined as schizophrenia, schizoaffective disorder, bipolar disorder or major depression) transitioning from jail, prison, or mental hospital settings to the community and found discharge planning with Medicaid-application assistance and integrated dual disorder treatment programs to be effective interventions to prevent recidivism.[22] Such planning is an important aspect of re-entry plans to help mentally ill inmates "connect with community-based mental health programs at the time of their release".[23] Draine, et al investigated mental health service records from a Medicaid database matched with admission to the Philadelphia jail system and reported that "49% of those with serious mental illnesses were released from jails through unpredictable release mechanisms, such as bail, release from court, or withdrawal of a bench warrant," and suggested that "re-entry programs for persons with mental illnesses should ensure that they have the capacity to rapidly identify and serve clients with shorter and more unpredictable jail stays".[24]

Implementation of the Affordable Care Act (ACA) has important implications for people with SMI released from incarceration because they tend to be single, part of a childless couple and therefore rarely covered by insurance. Of the approximately 10 million persons released from incarceration each year in the United States, about 70% to 90% were uninsured and the rate of mental illness, substance abuse disorders, and chronic health conditions in this group is about seven times higher than in the general population. The ACA is expected to provide coverage for about 35% of the schizophrenic population newly released from incarceration because the act provides Medicaid eligibility for anyone with income less than 138% (includes the 5% income disregard) of the federal poverty limit (FPL) regardless of the presence of children or disability. A particularly vulnerable population that will be provided benefits under ACA is a substantial portion of the homeless which includes many schizophrenics released from incarceration. Of adults in jail, 15.3% were homeless at some time in the previous year which is 7.5 to 11.3 times the estimate of homelessness in the general population.[25] The ACA does not require a "fixed address" residency requirement and the annual renewal process is automatic if there is no available information to disqualify a person.[26] However, many states have not elected to expand Medicaid coverage under ACA.

The magnitude of the relationship of Medicaid eligibility with risk of re-incarceration varied with the eligibility measure. Cumulative months of eligibility (eligibility 2) had an effect of about the same magnitude as age (HR = 0.978), while eligibility at the time of re-incarceration (eligibility 1) was associated with a larger decrease in likelihood of re-incarceration (HR = 0.659). The third eligibility variable, quarters of time to first eligibility since release from incarceration, is consistent with the literature cited earlier; increased time to obtaining Medicaid eligibility increases the hazard for re-incarceration (HR = 1.046). Of all the covariates (age, marital status, gender, race and substance abuse) only age was significant in the models estimated. These may have independent effects when not controlling for other effects. Their lack of significance in the presence of the three eligibility variables is further testimony to the importance of eligibility in the time to re-incarceration.

In addition to the relationship of Medicaid eligibility to re-incarceration we also studied the role of age. Each additional year of age was associated with a lower risk of incarceration (HR range of 0.986 to 0.988). This finding is in line with conventional wisdom, however, our results provide some estimate of the magnitude of the effect.

## CONCLUSIONS

All three eligibility measures in the 3-year models point to the same conclusion. Patients with eligibility at the point of re-incarceration or those with a greater number of cumulative months of eligibility have a lower risk of re-incarceration, while the greater number of quarters it takes to obtain eligibility is associated with a greater risk of re-incarceration. The results of this analysis suggest that Medicaid eligibility is an important mitigating factor for risk of re-incarceration of schizophrenia diagnosed persons.

## CONFLICT OF INTEREST DECLARATION

This study was supported by Janssen Scientific Affairs, LLC. The views expressed in this paper are those of the authors, and no official endorsement by Janssen Scientific Affairs, LLC, is intended or should be inferred.
